# Supplementary material for: Validity and reproducibility of a food frequency questionnaire to determine dietary intakes among Lebanese athletes
Source: PLoS One. 2024 Oct 23;19(10):e0311617. doi: 10.1371/journal.pone.0311617 (PMC11498687; doi:10.1371/journal.pone.0311617)
Supplement: Attachment — Submitted filename: PONE-D-24-19959_reviewer.pdf [file pone.0311617.s006.pdf]

PLOS ONE

Validity and reproducibility of a food frequency questionnaire to determine dietary intakes among Lebanese athletes

--Manuscript Draft--

Manuscript Number:	PONE-D-24-19959
Article Type:	Research Article
Full Title:	Validity and reproducibility of a food frequency questionnaire to determine dietary intakes among Lebanese athletes
Short Title:	Validity of a food frequency questionnaire among athletes
Corresponding Author:	Nagham Sannan, Ph.D. candidate<br>Saint Joseph University Faculty of Pharmacy: Universite Saint-Joseph Faculte de pharmacie<br>Beirut, LEBANON
Keywords:	Athletes; Food frequency questionnaire; Validity; Reproducibility; Nutrition; Dietary habits.
Abstract:	Background and objective: Nutrition is a basic need for athletes; thus, adequate dietary intake is crucial for maintaining overall health, facilitating training adaptations and boosting athletic performance. Accurate dietary assessment tools are required to minimize the challenges faced by athletes. This study verifies the validity and reproducibility of a 157 item semi-quantitative food frequency questionnaire among Lebanese athletes. This is the only Arabic questionnaire in Lebanon that estimates food consumption for athletes which can also be used in Arabic speaking countries. There has been no previous validated food frequency questionnaire that estimated food consumption for athletes in Lebanon.<br>Methods: A total of 194 athletes were included in the validation study and 81 of them also participated in the reproducibility phase after repeating the same food frequency questionnaire after one month, which was validated against four days dietary recalls (DRs).<br>Results: The intra-class correlation coefficient between the two-food frequency questionnaire ranged from 0.870 for energy (Kcal), 0.739 for carbs and 0.919 for proteins concerning macronutrients and from 0.688 for vitamin D, to 0.952 for vitamin B12, indicating an excellent reproducibility. The two dietary methods displayed a valid agreement using spearman's correlation coefficients, which varied between 0.304 for sodium, 0.469 for magnesium to 0.953 for caloric intake (kcal) and using Bland-Altman plots illustrated a percentage of agreement ranging between 94.3% for fats to 96.4% for proteins.<br>Conclusion: This FFQ has a reliable validity and reproducibility to evaluate dietary assessments and is an appropriate tool for future interventions to ensure the adoption of adequate eating strategies by athletes.
Order of Authors:	Nagham Sannan, Ph.D. candidate
	Tatiana Papazian
	Zeina Issa
	Nour El Helou
Additional Information:	
Question	Response
Financial Disclosure<br><br>Enter a financial disclosure statement that describes the sources of funding for the work included in this submission. Review the submission guidelines for detailed requirements. View published research	The author(s) received no specific funding for this work.

Powered by Editorial Manager® and ProduXion Manager® from Aries Systems Corporation

articles from *PLOS ONE* for specific examples.<br><br>This statement is required for submission and **will appear in the published article** if the submission is accepted. Please make sure it is accurate.<br><br>**Funded studies**<br>Enter a statement with the following details:<br>• Initials of the authors who received each award<br>• Grant numbers awarded to each author<br>• The full name of each funder<br>• URL of each funder website<br>• Did the sponsors or funders play any role in the study design, data collection and analysis, decision to publish, or preparation of the manuscript?<br><br>Did you receive funding for this work?	
**Competing Interests**<br><br>Use the instructions below to enter a competing interest statement for this submission. On behalf of all authors, disclose any competing interests that could be perceived to bias this work—acknowledging all financial support and any other relevant financial or non-financial competing interests.<br><br>This statement is required for submission and **will appear in the published article** if the submission is accepted. Please make sure it is accurate and that any funding sources listed in your Funding Information later in the submission form are also declared in your Financial Disclosure statement.<br><br>View published research articles from *PLOS ONE* for specific examples.	The authors have declared that no competing interests exist.

**NO authors have competing interests**<br><br>Enter: *The authors have declared that no competing interests exist.*<br><br>**Authors with competing interests**<br><br>Enter competing interest details beginning with this statement:<br><br>*I have read the journal's policy and the authors of this manuscript have the following competing interests: [insert competing interests here]*<br><br>* typeset	
**Ethics Statement**<br><br>Enter an ethics statement for this submission. This statement is required if the study involved:<br><br>• Human participants<br>• Human specimens or tissue<br>• Vertebrate animals or cephalopods<br>• Vertebrate embryos or tissues<br>• Field research<br><br>Write "N/A" if the submission does not require an ethics statement.<br><br>General guidance is provided below. Consult the submission guidelines for detailed instructions. **Make sure that all information entered here is included in the Methods section of the manuscript.**	The study followed the guidelines of the declaration of Helsinki and all procedures involving subjects were approved by the Research Ethics Committee of Saint Joseph University (USJ-2018-68).<br>Participants signed an informed written consent showing their agreement to take part in the study.

**Format for specific study types**<br><br>**Human Subject Research (involving human participants and/or tissue)**<br>• Give the name of the institutional review board or ethics committee that approved the study<br>• Include the approval number and/or a statement indicating approval of this research<br>• Indicate the form of consent obtained (written/oral) or the reason that consent was not obtained (e.g. the data were analyzed anonymously)<br><br>**Animal Research (involving vertebrate animals, embryos or tissues)**<br>• Provide the name of the Institutional Animal Care and Use Committee (IACUC) or other relevant ethics board that reviewed the study protocol, and indicate whether they approved this research or granted a formal waiver of ethical approval<br>• Include an approval number if one was obtained<br>• If the study involved *non-human primates*, add *additional details* about animal welfare and steps taken to ameliorate suffering<br>• If anesthesia, euthanasia, or any kind of animal sacrifice is part of the study, include briefly which substances and/or methods were applied<br><br>**Field Research**<br><br>Include the following details if this study involves the collection of plant, animal, or other materials from a natural setting:<br>• Field permit number<br>• Name of the institution or relevant body that granted permission	
**Data Availability**<br><br>Authors are required to make all data underlying the findings described fully available, without restriction, and from the time of publication. PLOS allows rare exceptions to address legal and ethical concerns. See the PLOS Data Policy and FAQ for detailed information.	Yes - all data are fully available without restriction

Powered by Editorial Manager® and ProduXion Manager® from Aries Systems Corporation

A Data Availability Statement describing where the data can be found is required at submission. Your answers to this question constitute the Data Availability Statement and **will be published in the article**, if accepted.<br><br>**Important:** Stating 'data available on request from the author' is not sufficient. If your data are only available upon request, select 'No' for the first question and explain your exceptional situation in the text box.<br><br>Do the authors confirm that all data underlying the findings described in their manuscript are fully available without restriction?	
**Describe where the data may be found in full sentences. If you are copying our sample text, replace any instances of XXX with the appropriate details.**<br><br>• If the data are **held or will be held in a public repository**, include URLs, accession numbers or DOIs. If this information will only be available after acceptance, indicate this by ticking the box below. For example: *All XXX files are available from the XXX database (accession number(s) XXX, XXX.)*.<br>• If the data are all contained **within the manuscript and/or Supporting Information files**, enter the following: *All relevant data are within the manuscript and its Supporting Information files.*<br>• If neither of these applies but you are able to provide **details of access elsewhere**, with or without limitations, please do so. For example:<br><br>*Data cannot be shared publicly because of [XXX]. Data are available from the XXX Institutional Data Access / Ethics Committee (contact via XXX) for researchers who meet the criteria for access to confidential data.*<br><br>*The data underlying the results presented in the study are available from (include the name of the third party*	All relevant data are within the manuscript and its Supporting Information files.

and contact information or URL).<br>• This text is appropriate if the data are owned by a third party and authors do not have permission to share the data.<br><br>* typeset	
Additional data availability information:	

Manuscript

Click here to access/download;Manuscript;Manuscript.docx

Validity and reproducibility of a food frequency questionnaire to determine dietary intakes among Lebanese athletes

Nagham Sannan[1*⁋], Tatiana Papazian[1⁋], Zeina Issa[1&], and Nour El Helou[1⁋]

[1] Laboratory of Human Nutrition, Nutrition Department, Faculty of Pharmacy, Saint Joseph University of Beirut, Beirut, Lebanon;

* Corresponding author

Email: nagham.sannan@net.usj.edu.lb

⁋ These authors contributed equally to this work.

& These authors also contributed equally to this work.

Abstract

Background and objective: Nutrition is a basic need for athletes; thus, adequate dietary intake is crucial for maintaining overall health, facilitating training adaptations and boosting athletic performance. Accurate dietary assessment tools are required to minimize the challenges faced by athletes. This study verifies the validity and reproducibility of a 157 item semi-quantitative food frequency questionnaire among Lebanese athletes. This is the only Arabic questionnaire in Lebanon that estimates food consumption for athletes which can also be used in Arabic speaking countries. There has been no previous validated food frequency questionnaire that estimated food consumption for athletes in Lebanon.

Methods: A total of 194 athletes were included in the validation study and 81 of them also participated in the reproducibility phase after repeating the same food frequency questionnaire after one month, which was validated against four days dietary recalls (DRs).

Results: The intra-class correlation coefficient between the two-food frequency questionnaire ranged from 0.870 for energy (Kcal), 0.739 for carbs and 0.919 for proteins concerning macronutrients and from 0.688 for vitamin D, to 0.952 for vitamin B12, indicating an excellent reproducibility. The two dietary methods displayed a valid agreement using spearman's correlation coefficients, which varied between 0.304 for sodium, 0.469 for magnesium to 0.953 for caloric intake (kcal) and using Bland-Altman plots illustrated a percentage of agreement ranging between 94.3% for fats to 96.4% for proteins.

Conclusion: This FFQ has a reliable validity and reproducibility to evaluate dietary assessments and is an appropriate tool for future interventions to ensure the adoption of adequate eating strategies by athletes.

Keywords: Athletes, Food frequency questionnaire, Validity, Reproducibility, Nutrition, Dietary habits.

Introduction

Sports nutrition is a modern discipline that involves the implementation of nutritional fundamentals in order to improve athletic performance [1]. Optimal nutrition delays fatigue, prolongs training durations and allows faster recovery between training sessions [2]. Thus, nutrition is an essential component of any physical activity, and that is the reason why active individuals strive to follow adequate dietary guidelines for maximum health optimization and sports performance [3]. Athletes' adherence to training programs and eating strategies produce optimal performance during competitions and quick recovery after exercise sessions [4, 5]. Moreover, dietary interventions through proper nutrient and food intake estimations stimulate overall health and competitive performance among athletes [6, 7]. Appropriate nutritional advice and counseling are essential to understand this population's knowledge. In epidemiological studies, distinctive methods are used to determine individuals' nutritional intake [8, 9]; the food frequency questionnaire (FFQ) is a low expense tool that is widely adopted in these studies among a large population [10]. The analysis of FFQs as beneficial methods has contributed in estimating dietary intakes in equitable measurements of energy, macronutrients, micronutrients, and fluids in various subgroups of the population worldwide [11]. Since dietary habits vary greatly depending on the ethnic, social and cultural background of the specified participants, FFQs must be tailored and validated to target specific populations because the food items should reflect their dietary habits [12]. In 2016, Lebanon has

witnessed the validation of two FFQs, one among pregnant women [13] and the other among children [14]. Two other FFQs were validated in 2019 among Lebanese adults respectively [15, 16]. Since FFQs can serve as a valid and accurate tool for assessing the nutritional intakes of athletes [17], the purpose of this research is to validate a previously developed FFQ among Lebanese athletes.

Materials and methods

2.1 Study population

Our study sample comes from the sports teams of Lebanese Military organizations represented by sports teams of the Lebanese Army and Internal Security Forces where these organizations have teams of professional athletes representing all the geographical areas of Lebanon. These teams are dedicated to sports and they are rigorously trained for participation in national and international competitions and also represent the country in these events. Based on this, and after acquiring the necessary authorizations from these organizations, we were provided with the lists of names of 255 athletes from 18 different teams and that come from all Lebanese geographical districts that included the capital Beirut and regions in Mount Lebanon, North Lebanon, South Lebanon and the Beqaa. After these athletes were contacted by phone and invited to participate in our study, a total of 200 athletes agreed to participate. The study protocol was approved by the Institutional Review Board of Saint-Joseph University of Beirut and its Ethics Committee (USJ-2018-68) and was conducted according to the declaration of Helsinki guidelines. After an informative session prior to the study, all participants gave their written consent to participate in our study. The study was conducted between May 14, 2019 and March

11, 2020 by trained dietitians and face to face interviews. The athletes included in our study had to meet inclusion criteria that require them to be Lebanese, between the ages of 18 and 50, previous participants in Lebanese Championships for at least the three previous years as competent athletes, and must not have any medical conditions or use any type of medications that might influence their food intake. One hundred ninety-four participants were included in the final pool of analysis.

2.2 Development of the FFQ

The quantitative 157-item FFQ was developed by a panel of nutritionists from the faculty staff at the nutrition department of the faculty of pharmacy at Saint-Joseph University of Beirut (USJ) and was previously tested, in a pilot study, during a face-to-face interview on 50 athletes from the sample [12,13]. It was divided into 12 food sections, with ingredients highly representative of the Lebanese cuisine, as follows: cereals and bread, rice and grains, milk and dairy products, fruits and juices, vegetables, meats and alternatives, nuts, sweets, bakeries, salty snacks, oils, fats, and finally beverages and drinks, including alcohol [18]. The number and frequency of each portion consumed was recorded on a daily, weekly, monthly, or yearly basis [19]. Seasonal fruit items were also taken into account while determining the frequency of their consumption throughout the period of the year [19]. In order to calculate the total amount of daily food intake (Kcal/day), the frequency of consumption was converted to times per day and then multiplied by its reported portion size [20]. This study adapted and validated the FFQ for athletes knowing that it was previously validated among the Lebanese adult population [13, 15]. The total duration for filling the FFQ was estimated to be around 40 minutes

[21]. Expert dietitians completed face to face interviews where each participant was asked specific questions about his/her food routine. To simplify the participants' answers, we used standard local household units such as plates, bowls, and spoons of different sizes, plastic food models and food photos. The same FFQ was administered at baseline (FFQ1) and one month later (FFQ2).

2.3. Methodology

In order to ensure complete validation of the FFQ, we underwent the average procedure of collecting a four day 24h dietary recalls [DRs] as a suitable compromise between the scientific vigor and the practicability, for the purpose of determining energy, macronutrient and micronutrient intakes. Hence, interviews were planned on weekdays and weekends to detect intake distinction among the participants. These participants filled 24h DR of two days repeated twice within a one-month interval with the intention of obtaining a total of four non-consecutive 24h DRs [22]. In addition to this, the research team took the anthropometric measurements and filled basic socio-demographic and lifestyle questionnaires of all the participants. In a systematic review, Cade et al. (2002) claimed that the 24h DRs questionnaire are used as an accurate reference method against FFQs [12]. For this reason, the validity of the FFQ was determined by comparing energy and nutrient intakes estimated from the FFQ and the average of the four-day DRs. Flowcharts in "Fig 1" provide a representation of this information. This method guarantees less measurement errors and more reliable and credible associations [23]. Moreover, the five-step multiple-pass method declared by the United States Department of Agriculture was performed [24]. The primary reason for the use of this method is its

accuracy and ease of food recall by the participants. The specific steps begin with an immediate list of foods consumed, potentially forgotten foods, followed by recording the meal times/ occasion for precision, then giving detailed quantities and ingredients, and a final review. Overall, participants needed 25-30 minutes to complete each 24h DR, accordingly with the recommendations [25].

The sample size chosen to test reproducibility was deliberately selected according to researchers in the field Cade et al. (2010) and Willet et al. (2012) who recommended a minimum percentage of 30% of the initial sample randomly chosen [8,12]. In order to alleviate possible biases, questionnaires were filled by the same interviewer for all the athletes [22]. Interview-based questionnaires lead to faster completion and more consistent analysis compared to self-administered questionnaires [26]. To achieve the reproducibility of our tested tool, the nutritionists completed a second FFQ with the participants, one month later. The reproducibility of the FFQ was conducted among 81 participants who completed the questionnaire twice by the dietitians within a 1-month time interval [25]. This interval was adequate for the participants to forget their previous responses, and was considered as a safe period to detect any significant and tangible changes in their dietary habits [12]. It should also be mentioned that exceeding a one-month interval might cause seasonal reporting biases [27].

2.4 Nutrient intake calculation

In order to process all the information provided by the FFQ, frequencies were converted into daily intake in grams. After that, analysis of the FFQ and the 24h DRs and the estimation of energy and nutrient intakes were performed respectively using Nutrilog®

2.3 software. The United States Department of Agriculture (USDA) database and the French food composition table CIQUAL provide nutrient values for all the international food items [28, 29]. Concerning Lebanese food recipes, the database of the American University of Beirut was used [30]. All three mentioned databases were incorporated within the Nutrilog® software.

2.4.1 Relative validity of the food frequency questionnaire.

In order to accurately track energy and nutrient intakes for the FFQ and the four 24h DRs, means and standard deviations were calculated. The uniformity of the distribution was evaluated based on the usage of Kolmogorov-Smirnov test. The majority of nutrients underwent a skewed distribution, hence non-parametric tests were conducted. Variables of FFQs and 24h DRs were compared using the Wilcoxon signed-rank test to examine absolute differences between the 24h DRs and the FFQ1 and FFQ2 [31]. Furthermore, for the purpose of assessing an agreement between the two tools for total energy intake, macronutrients, and micronutrients, Bland Altman Plots were implemented. Spearman's correlation coefficient was used to test the reproducibility of the FFQ and the relationships between estimated macronutrients and micronutrients between the FFQs and 24h DRs [32].

2.4.2 Reproducibility of the food frequency questionnaire.

Nutrient intakes of both FFQs (FFQ1 and FFQ2) were assessed based on means and standard deviations. To measure the reproducibility of the FFQ, intra-class correlation coefficients (ICC) were calculated between values attained in the two FFQs. It was then evaluated using the following classification: weak reliability (<0.5), moderate reliability

(0.5 to 0.75), good reliability (0.75 to 0.90), and excellent reliability (>0.9). The SPSS statistical software was applied to complete all statistical analyses (SPSS for windows, version 24.0, USA). A p-value less than 0.05 was interpreted as significant [33].

Results

3.1. Socio-demographic and anthropometric characteristics of the population

The final sample included one hundred and ninety-four athletes, as shown in table 1; the majority of the participants are males and their age range were between 21 and 47 years old with a mean of 30.05 years. Regarding the educational level, more than half of the population achieved secondary education or above and this is considered an acceptable academic level to have basic information and common knowledge about their food intakes and portion sizes. Furthermore, the majority of the population consumes two to three meals a day (62.4%).

Table1.Characteristics of the study sample (n=194).

Characteristic		***Frequency***	***Percentage***
Gender	***Male***	183	94.30%
	Female	11	5.70%
Civil status	***Single***	85	43.80%
	Married	22	11.30%
	Engaged	84	43.30%
	Divorced	2	1.00%
	Widow	1	0.50%
Self- reported perceived financial status*	***Good***	20	10.30%
	Medium	94	48.50%
	Careful with expenses	44	22.70%
	Critical	27	13.90%
	No answer	9	4.60%
Level of education	***No education***	1	0.50%
	Primary	25	12.90%
	Lower Secondary	71	36.60%
	Secondary	29	14.90%
	Baccalaureate	43	22.20%
	Bachelor Degree	22	11.30%
	Master Degree	1	0.50%
	No answer	2	1.00%
Number of meals consumed per day	***1-2***	34	17.50%
	2-3	121	62.40%
	4-5	39	20.10%

*****Prior to the 2019 Lebanese financial crisis***

3.2. Reproducibility of the food-frequency questionnaire

The reproducibility study involved eighty-one athletes. The results presented in table 2, showed an ICC between the two FFQ measurements that ranged between 0.870 for energy (Kcal), 0.739 for carbs to 0.919 for proteins of macronutrients, 0.688 for vitamin D, 0.804 for vitamin B6, 0.952 for vitamin B12, 0.940 for sugar, and 0.917 for polyunsaturated fatty acids. These results indicate excellent reproducibility of the tested tool.

Table2. Reproducibility study: Mean daily energy and nutrient intakes and the intra-class coefficient for the comparison between FFQ1 and FFQ2 in Lebanese athletes.

Parameters	*FFQ1 Mean ± SD*	*FFQ2 Mean ± SD*	*ICC (with 95% CI)*
Energy (kcal)	2526.72±587.68	2707.57±527.77	0.870 [0.798- 0.916]
Carbs (%)	55.81±10.00	57.80±10.26	0.857 [0.778- 0.908]
Carbs(g)	4.46±1.55	6.21±2.21	0.739 [0.594- 0.832]
Sugars (g)	27.29±8.06	30.82±9.12	0.940 [0.906- 0.961]
Fat (%)	32.19±7.53	35.60±6.70	0.825 [0.728- 0.887]
Polyunsaturated Fatty Acids (g)	18.63±6.11	18.01±8.18	0.917 [0.871- 0.947]
Proteins (%)	20.32±5.10	22.89±5.31	0.889 [0.827- 0.929]
Proteins (g)	1.61±0.59	1.74±0.61	0.919 [0.874- 0.948]
Vitamin A(µg)	311.16±224.68	317.85±201.29	0.885 [0.821- 0.926]
Vitamin D (mg)	11.49±4.19	10.79±2.73	0.688 [0.515- 0.799]
Vitamin E (mg)	11.11±5.33	11.78±5.67	0.942 [0.909- 0.962]
Vitamin C (mg)	88.11±46.23	103.24±47.93	0.930 [0.892- 0.955]

Vitamin B6 (mg)	2.55±0.81	2.69±0.93	0.804 [0.696- 0.874]
Folate (µg)	304.54±174.21	301.27±149.51	0.873 [0.802- 0.918]
Vitamin B12 (mg)	7.55±7.63	6.49±7.90	0.952 [0.925- 0.969]
Magnesium (mg)	377.56±153.68	384.12±159.31	0.910 [0.860- 0.942]
Calcium (mg)	935.10±296.46	976.84±258.59	0.905 [0.853- 0.939]
Potassium (mg)	3669.40±1327.78	3579.78±1359.59	0.897 [0.840- 0.934]
Sodium (mg)	6022.15±1345.10	6189.49±1445.99	0.949 [0.921- 0.967]
Iron (mg)	25.02±7.81	22.96±9.55	0.844 [0.757- 0.899]
Zinc (mg)	11.59±5.11	11.39±5.02	0.877 [0.809- 0.921]
Selenium (mg)	132.74±46.79	135.87±50.92	0.906 [0.854- 0.939]

3.3 Relative validity of the food-frequency questionnaire

The validation of the FFQ was assessed by comparing the intakes of energy, macronutrients, and micronutrients to four 24h DRs [34, 35] .Table 3 presents descriptive statistics and spearman correlations between nutrient intakes derived from the FFQ and the 24h DRs showing that FFQ's intakes are higher than 24h DRs ranging from 5.6% for proteins, 7.8% for fat, 8% for energy, 47.2% for vitamin E, and 60.6% for vitamin B12. According to the normality test, all variables in this case are skewed; hence, Wilcoxon signed rank test was executed and demonstrated a significant difference between the means of DRs and FFQ ($p<0.05$). According to Spearman's test, there is a significant correlation between the FFQ and 24h DRs with a coefficient ranging between 0.304 for sodium, 0.469 for magnesium, 0.686 for vitamin A, 0.722 for carbohydrates (g/kg) and 0.953 for energy which is considered as a positive correlation ranging from weak to high.

Cross-classification analysis revealed that about 92.8% of the participants were in the same quartile for vitamin A and 94.3% for polyunsaturated fatty acids.

Table 3. Validation study: FFQ and four 24h DRs. Comparison of mean daily intake of energy and nutrients.

Parameters	***24h DR Mean ± SD***	***FFQ Mean ± SD***	***p-value*** [a]	***Relative Difference (%)*** [b]	***Correlation Coefficient*** [c]
Energy (kcal)	2359.20±597.48	2548.55±667.42	<0.001	8.03%	0.953
Carbohydrates (%)	49.81±8.58	54.75±9.72	<0.001	9.92%	0.868
Carbohydrates (g/kg)	3.78±1.29	4.13±1.62	<0.001	9.35%	0.722
Sugar (g)	25.40±9.62	28.03±7.81	<0.001	10.35%	0.511
Fats (%)	30.38±6.25	32.76±7.58	<0.001	7.84%	0.625
Polyunsaturated fatty acids(g)	12.55±5.83	19.23±6.04	<0.001	53.22%	0.573
Proteins (%)	19.74±5.14	20.85±6.19	0.002	5.63%	0.574
Proteins (g/kg)	1.48±0.55	1.58±0.58	<0.001	6.56%	0.636
Vitamin A (μg)	253.29±284.57	335.71±228.93	<0.001	32.54%	0.686
Vitamin D (μg)	9.61±2.59	11.21±3.90	<0.001	16.55%	0.331
Vitamin E (mg)	7.66±6.20	11.28±6.22	<0.001	47.21%	0.643
Vitamin C (mg)	87.58±58.78	102.25±60.65	<0.001	16.74%	0.776
Vitamin B6 (mg)	2.02±1.15	2.62±1.05	<0.001	29.25%	0.809
Folate (μg)	290.18±149.64	314.20±180.01	0.001	8.28%	0.720
Vitamin B12 (mg)	4.94±12.45	7.93±12.35	<0.001	60.56%	0.557
Magnesium (mg)	309.00±132.67	377.63±163.13	<0.001	22.21%	0.469

Calcium (mg)	667.49±300.90	946.07±309.24	<0.001	41.74%	0.637
Potassium (mg)	2905.06±1065.72	3580.19±1326.72	<0.001	23.24%	0.426
Sodium (mg)	4701.94±1161.34	6002.03±1409.63	<0.001	27.65%	0.304
Iron (mg)	16.57±6.47	25.06±9.37	<0.001	51.26%	0.353
Zinc (mg)	8.75±4.42	10.84±5.38	<0.001	23.82%	0.542
Selenium (µg)	102.76±72.88	140.10±65.44	<0.001	36.33%	0.776

[a] Wilcoxon signed rank test were used to compare values obtained from both FFQ and 4-24h DRs

[b] Relative difference = [(FFQ– 24h DR)/24h DR]x100.

[c] Spearman correlation coefficient were used to assess the correlation between variables with a p-value < 0.05 considered as significant

3.4. Bland-Altman analysis

The graphical representation Bland-Altman plot illustrates the study of the agreement of the nutrients derived from the FFQ and the 24h DRs. The average difference between the two methods (FFQ and 24h DRs) is represented by the solid line, while the distance between the mean of the difference ± two standard deviations is designated by the hatched lines. The variation between positive and negative values demonstrated that nutritional intakes were both over and under estimated by the FFQ compared to 24h DRs which is considered to be the limit of agreement. The Bland-Altman analysis of energy, carbohydrates, fats, proteins, iron, and fatty acid intake is represented in “Fig 2” with a homogeneous dispersion above and below zero in most plots. The Bland-Altman method has proved to be the most effective in determining absolute validity by estimating mean

agreement and the limits of agreement. Mean agreement indicates the average of the difference between the assessment tools that aid in determining food intake [36, 37]. This particular value was almost the same with the two assessment tools (the FFQ and the 24h DR), which shows a good degree of absolute validity. As for the nutrient intakes, the Bland- Altman analysis illustrated a percentage of agreement ranging between 94.3% for fats to 96.4% for proteins.

Discussion

The objectives of this study were to examine the validity and the reproducibility of the FFQ administered among a group of Lebanese athletes. The tested tool has proved to be the most practical and cost-effective method in order to assess diets in large-scale nutritional epidemiological studies [38]. To our knowledge, this is the first study validating culture-driven tool for Lebanese athletes providing an estimate of their nutritional habits. The aim of the FFQ is to establish a direct relationship between food intake and the athletes' condition which is highly affected by the quality and quantity of their meals [39].

Literature reviews show that FFQ's validity is assessed by comparing it to a 24h DR in all epidemiological studies [40]. Nevertheless, some authors would prefer the collection of at least two 24h DRs due to the fact that this type of recall relies immensely on memory and is influenced by day-to-day and seasonal variation which is distributed along a definite time frame. Hence, in order to be more accurate and alleviate bias, four 24h DRs were collected for each athlete by the same interviewer [41]. It must be noted that the one-month interval between repeated measurements is sufficient to reduce dietary changes over time as well as the memory of previous answers [12; 42]. As a matter of

fact, a time interval more than one month and up to three months could lead to the emergence of a seasonality bias [43]. Despite this, previous studies had time intervals of two weeks [44], three weeks [45, 46], four weeks [47, 48], four to six weeks [49] and six weeks [50, 51]. Validity was assessed using correlation coefficients (CCs) by comparing energy and nutrient intakes estimated from the FFQ and the average of the four-day DRs [52, 53]. According to Willet (1998) and Cade et al. (2002), the best obtainable values for CCs in dietary validation studies are between 0.5 and 0.7. In our study, the correlation coefficients were above 0.5 for the majority of nutrients: 0.953 for energy (kcal), 0.868 for carbohydrates, 0.625 for fats (%), 0.574 for proteins (%), 0.776 for vitamin C and 0.686 for vitamin A. Our data indicate a relatively high correlation level between the FFQ and the reference method. In comparison with many other studies, a few have been performed on athletes; thus, this study shows CC values within a successful range (0.636 of proteins (g/kg) →0.953 energy) regarding macronutrients and (0.3→0.809) regarding micronutrients. According to Morel et al. in a French-Canadian study conducted in 2018, the values ranged between 0.56 and 0.88 [54]. Moreover, another study published by Fallaize et al. (2014) showed that the CCs ranged between 0.65 and 0.9; which are considered to be substantially higher than those found in an earlier study [55]. To further ensure the highly acceptable CCs in our own study , two other examples were taken into consideration; the first one involved Cheng et al.(2020) that recorded a CC of 0.63 for carbohydrates, 0.21 for fats, and 0.34 for proteins, 0.62 for vitamin C and 0.55 for vitamin A [56]. The second study by Sasaki et al. (2018) noted a median CC of 0.3 for males and 0.32 for females [57]. As a result, our range has proved to have a higher CC than in those two mentioned studies. In our study, there are some factors that have

contributed to the high validity and correlation compared to others. First, the comprehensive food list positively affected the results. Second, the method of administration of the questionnaire in which data is collected by a professional dietitian with the same exact athlete mitigated the variability. Third, the personal interview presented spontaneous and accurate responses from the participants. Finally, the type of questions facilitated effective communication and contributed to the validity of the results. Moreover, stronger correlations were withheld upon the exclusions of participants who tend to either under or overestimate their total energy intakes, and who suffer from a certain health condition that could have an impact on their nutritional intake or health status. As for the arrangement of the intake levels, the quartile notation was most suitable; this study is appropriate and compatible with results previously reported by Zaragoza et al. (2017) in which more than 84% of the subjects were grouped in the same quartile [58]. It is noted that there was a consistent overestimation of the FFQ to most of the nutrient intakes reaching 5.6% for protein, 7.81% for fats, 47.2% for vitamin E and 60.6% for vitamin B12 with a difference of 8% for energy. This variation between the FFQ and 24h DRs could be mainly due to the fact that athletes tend to eat bigger portions and each one of them focuses on his food intake depending on the season and competitions. Another possible reason for this overestimation is the number of food items that makes it difficult for participants to recall their exact yearly consumption while filling the FFQ with respect to the 24h DRs [48,59] as Pinto et al.(2010) proposed that this overestimation may be due to difficulties in accurately estimating the portion size consumed [60;61].

Consequently, the Bland Altman analyses were performed in order to demonstrate the compliance between the FFQ and the 24h DRs by showing the difference between the two methods against their averages. A mean of differences that is close to zero implies a more comprehensive agreement between the two tools. Hence, this FFQ displayed a very good validity compared to the 24h DRs [62].

The reproducibility of the FFQ was excellent since the ICC from this study ranged from 0.825 to 0.889 for macronutrients and 0.688 to 0.952 for micronutrients. Compared to other studies, Ishikawa-Takata et al.'s (2021) results for Japanese athletes showed an ICC of 0.588 for carbohydrate, 0.619 for energy and 0.752 for vitamins [63]. Moreover, a study of the validity and reproducibility of a culture–specific FFQ developed in Lebanon demonstrated an ICC of 0.44 for protein, 0.67 for fat and 0.85 for energy [32]. As a result, these observations indicate that our FFQ has a high reproducibility; this might be explained by the fact that these athletes included in our study are subject to regular nutritional follow ups in a structured environment controlled by professional sport dietitians.

Strengths and limitations

We acknowledge some inevitable limitations concerning this research. The extended list of 157 food items might have biased the answers of the participants and have led to overestimations [64]. As for the validation of the FFQ, the 24h DR was chosen to be the reference method which could be seen a possible weakness since both the FFQ and the 24h DR have related measurement errors including memory bias, misreporting of portion sizes and cooking techniques, and other seasonal variations [65, 66]. In addition, the

heterogeneity of the sample of athletes, derived from 18 professional sports teams, can influence the results. A serious potential error that is common to self-reported dietary methods is the estimation of nutrients based on food composition tables. The nutritional composition of food varies depending on the season, location of production, growing conditions, storage, processing and cooking techniques. A lot of these mentioned factors are not taken into consideration in the food composition tables. It must be noted that determining the nutritional composition of food intake is a significant challenge, which is the main reason to continuously work towards improving the accuracy of food composition databases.

On the other hand, it is worth mentioning that the current study's major strength was the comprehensive approach utilized to establish the FFQ items that are suitable and in line with the food culture of the Lebanese athletes. The fact that the FFQ was interviewer-administered and not self-administered improved accuracy by allowing for immediate clarification of any misunderstandings and reduced inaccuracies. It also allowed more control by ensuring standardized portion sizes and intake frequencies and reducing the risk of misinterpretations by participants. This FFQ includes food items that are related to the Mediterranean culture in the Arab world, making it a reliable tool in Arabic language that can be used by Arab-speaking athletes in the Arab world. Moreover, the sample size exceeds the average number of participants in other validation studies since our study had a high participation rate. Another strength for this study is the statistical methodology applied. A recent review conducted by Lombard et al. (2015) claimed that the percentage of statistical tests usually used in validation studies range between one and three. Nevertheless, we applied all appropriate methods while developing a methodological

framework that is considered adequate to transmit logical insights into various factors of validity. Thus, when various statistical analyses are administered, a better reproducibility and validity of our tools are found vividly.

When it comes to critical research issues, time constraints, financial factors, and participant burdens are clearly found which is why future investigations in this particular field should develop online questionnaires for a better assessment of the nutritional intakes. Also, using computerized systems in epidemiology has the ability to favor the study by permitting faster responses, mitigating the bias of incomplete data using automated in controls which eases the estimation of food portions through illustrative models.

Conclusion

The purpose of this study was to adopt a 157-item semi-quantitative FFQ that was already developed and validated among Lebanese adults and Lebanese pregnant women, to evaluate its validity and reproducibility among Lebanese Athletes. In conclusion, we attempt to offer a valid and reliable FFQ that would be beneficial in estimating nutrient intakes for the purpose of developing appropriate strategies to progress the diets of the athletes needing a thorough investigation of their dietary habits. This particular FFQ is also appropriate for trials that assess the impact of nutritional interventions on dietary practices. In order to limit the FFQ's administration time and to mitigate the length of the listed items given that they tend to cause confusion and overestimation of consumption among athletes, future work and focus must be done to develop a smaller yet efficient computerized version of this FFQ, taking into consideration the special methodological

considerations for athletes including serving sizes, having snacks, water and beverage consumption, supplement use and weight control.

Supporting information

S1 File. Data. (XLSX)

S2 File. Data (XLSX)

S3 File. FFQ questionnaire (DOC X)

Acknowledgments

The authors would like to thank all the participants whose contribution helped in the success of this research. We would also like to thank Mrs. Hanine Abou Antoun for her contribution and help in statistics and Mrs. Steffi Younes for her contribution to the research.

Author Contributions

Conceptualization: Nagham Sannan, Nour El Helou.

Data curation: Nagham Sannan, Zeina Issa, Nour El Helou.

Formal Analysis: Nagham Sannan, Nour El Helou.

Investigation: Nagham Sannan, Tatiana Papazian, Zeina Issa, Nour El Helou.

Methodology: Nagham Sannan, Tatiana Papazian, Zeina Issa, Nour El Helou.

Project administration: Nagham Sannan, Nour El Helou.

Supervision: Nour El Helou.

Validation: Nagham Sannan, Tatiana Papazian, Zeina Issa, Nour El Helou.

Writing-original draft: Nagham Sannan.

Writing-review and editing: Nagham Sannan, Tatiana Papazian, Nour El Helou.

Declaration of interest statement

The authors declare no conflict of interest.

Consent for publication

All authors read and approved the final manuscript and agreed for its publication.

References

1. Ozdoğan Y, Ozcelik AO. Evaluation of the nutrition knowledge of sports department students of universities. J Int Soc Sports Nutr. 2011;8(1). Available from: http://dx.doi.org/10.1186/1550-2783-8-11

2. Capling L, Beck K, Gifford J, Slater G, Flood V, O'Connor H. Validity of dietary assessment in athletes: A systematic review. Nutrients. 2017;9(12):1313. Available from: http://dx.doi.org/10.3390/nu9121313

3. Vázquez-Espino K, Rodas-Font G, Farran-Codina A. Sport nutrition knowledge, attitudes, sources of information, and dietary habits of sport-team athletes. Nutrients. 2022;14(7):1345. Available from: http://dx.doi.org/10.3390/nu14071345

4. Heaton LE, Davis JK, Rawson ES, Nuccio RP, Witard OC, Stein KW, et al. Selected in-season nutritional strategies to enhance recovery for team sport athletes: A practical overview. Sports Med. 2017;47(11):2201–18. Available from: http://dx.doi.org/10.1007/s40279-017-0759-2

5. Hornstrom GR, Friesen CA, Ellery JE, Pike K. Nutrition knowledge, practices, attitudes, and information sources of mid-American conference college softball players. Food Nutr Sci. 2011;02(02):109–17. Available from: http://dx.doi.org/10.4236/fns.2011.22015

6. U.S. Sports Academy. The Sport Journal. 2008. Nutrition-related knowledge, attitude, and dietary intake of college track athletes. Available from: https://thesportjournal.org/article/nutrition-related-knowledge-attitude-and-dietary-intake-of-college-track-athletes/

7. Molina-López J, Molina JM, Chirosa LJ, Florea D, Sáez L, Jiménez J, et al. Implementation of a nutrition education program in a handball team; consequences on nutritional status. Nutr Hosp. 2013;28(4):1065–76. Available from: http://dx.doi.org/10.3305/nh.2013.28.4.6600

8. Willett WC. Nutritional Epidemiology. 3rd ed. New York, NY: Oxford University Press; 2012.

9. Denova-Gutiérrez E, Ramírez-Silva I, Rodríguez-Ramírez S, Jiménez-Aguilar A, Shamah-Levy T, Rivera-Dommarco JA. Validity of a food frequency questionnaire to assess food intake in Mexican adolescent and adult population. Salud Publica Mex. 2016;58(6):617. Available from: http://dx.doi.org/10.21149/spm.v58i6.7862

10. Hebden L, Kostan E, O'Leary F, Hodge A, Allman-Farinelli M. Validity and reproducibility of a food frequency questionnaire as a measure of recent dietary intake in young adults. PLoS One. 2013;8(9): e75156. Available from: http://dx.doi.org/10.1371/journal.pone.0075156

11. Thompson FE, Subar AF. Dietary assessment methodology. In: Nutrition in the Prevention and Treatment of Disease. Elsevier; 2017. p. 5–48.

12. Cade J, Thompson R, Burley V, Warm D. Development, validation and utilisation of food-frequency questionnaires – a review. Public Health Nutr. 2002;5(4):567–87. Available from: http://dx.doi.org/10.1079/phn2001318

13. Papazian T, Hout H, Sibai D, Helou N, Younes H, El Osta N, et al. Development, reproducibility and validity of a food frequency questionnaire among pregnant women adherent to the Mediterranean dietary pattern. Clin Nutr. 2016;35(6):1550–6. Available from: http://dx.doi.org/10.1016/j.clnu.2016.04.015

14. Moghames P, Hammami N, Hwalla N, Yazbeck N, Shoaib H, Nasreddine L, et al. Validity and reliability of a food frequency questionnaire to estimate dietary intake among Lebanese children. Nutr J. 2015;15(1). Available from: http://dx.doi.org/10.1186/s12937-015-0121-1

15. Aoun C, Bou Daher R, El Osta N, Papazian T, Khabbaz LR. Reproducibility and relative validity of a food frequency questionnaire to assess dietary intake of adults living in a Mediterranean country. PLoS One. 2019;14(6): e0218541. Available from: http://dx.doi.org/10.1371/journal.pone.0218541

16. Harmouche-Karaki M, Mahfouz M, Obeyd J, Salameh P, Mahfouz Y, Helou K. Development and validation of a quantitative food frequency questionnaire to assess dietary intake among Lebanese adults. Nutr J. 2020;19(1). Available from: http://dx.doi.org/10.1186/s12937-020-00581-5

17. Sharma S. Development and use of FFQ among adults in diverse settings across the globe. Proc Nutr Soc. 2011;70(2):232–51. Available from:

http://dx.doi.org/10.1017/s0029665110004775

18. WHO Regional Office for the Eastern Mediterranean. Promoting a healthy diet for the WHO eastern Mediterranean Region: User-friendly guide. Genève, Switzerland: World Health Organization; 2012.

19. Biró G, Hulshof K, Ovesen L, Amorim Cruz JA, for the EFCOSUM Group. Selection of methodology to assess food intake. Eur J Clin Nutr. 2002;56(S2): S25–32. Available from: http://dx.doi.org/10.1038/sj.ejcn.1601426

20. Athanasiadou E, Kyrkou C, Fotiou M, Tsakoumaki F, Dimitropoulou A, Polychroniadou E, et al. Development and validation of a Mediterranean oriented culture-specific semi-quantitative food frequency questionnaire. Nutrients. 2016;8(9):522. Available from: http://dx.doi.org/10.3390/nu8090522

21. Yaghi N, Boulos C, Baddoura R, Abifadel M, Yaghi C. Validity and reliability of a food frequency questionnaire for community dwelling older adults in a Mediterranean country: Lebanon. Nutr J. 2022;21(1). Available from: http://dx.doi.org/10.1186/s12937-022-00788-8

22. Yuan C, Spiegelman D, Rimm EB, Rosner BA, Stampfer MJ, Barnett JB, et al. Validity of a dietary questionnaire assessed by comparison with multiple weighed dietary records or 24-hour recalls. Am J Epidemiol. 2017;185(7):570–84. Available from: http://dx.doi.org/10.1093/aje/kww104

23. Mitry P, Wawro N, Six-Merker J, Zoller D, Jourdan C, Meisinger C, et al. Usual dietary intake estimation based on a combination of repeated 24-H food lists and a food frequency questionnaire in the KORA FF4 cross-sectional study. Front Nutr. 2019;6. Available from: http://dx.doi.org/10.3389/fnut.2019.00145

24. Usda.gov. AMPM - USDA automated multiple-pass method: USDA ARS. Available from: https://www.ars.usda.gov/northeast-area/beltsville-md-bhnrc/beltsville-human-nutrition-research-center/food-surveys-research-group/docs/ampm-usda-automated-multiple-pass-method/

25. Baranowski T. 24-hour recall and diet records methods. Nutr Epidemiol Third edition. 2012;

26. Tayyem RF, Albataineh SR, Allehdan S, Badran E. Relative validity and

reproducibility of a food frequency questionnaire to evaluate food group intakes among Jordanian children and adolescents. Nutr Hosp. 2021;38(5):1047–57. Available from: http://dx.doi.org/10.20960/nh.03528

27. Fowke JH, Schlundt D, Gong Y, Jin F, Shu XO, Wen W, et al. Impact of season of food frequency questionnaire administration on dietary reporting. Ann Epidemiol. 2004;14(10):778–85. Available from: http://dx.doi.org/10.1016/j.annepidem.2004.02.002

28. Department of Agriculture, Agricultural Research Service. USDA National Nutrient Database for Standard Reference. SR 23. 2010.

29. The French Agency for Food, Environmental and Occup Health Saf (ANSES). Ciqual French Food Composition Table version. 2008.

30. Pellet PL, Shadarevian S. Food composition tables for use in the Middle East. Beirut: American University of Beirut; 1970.

31. Zang J, Luo B, Chang S, Jin S, Shan C, Ma L, et al. Validity and reliability of a food frequency questionnaire for assessing dietary intake among Shanghai residents. Nutr J. 2019;18(1). Available from: http://dx.doi.org/10.1186/s12937-019-0454-2

32. El Sayed Ahmad R, Baroudi M, Shatila H, Nasreddine L, Chokor FAZ, Chehab RF, et al. Validity and reproducibility of a culture-specific food frequency questionnaire in Lebanon. Nutrients. 2020;12(11):3316. Available from: http://dx.doi.org/10.3390/nu12113316

33. Lombard MJ, Steyn NP, Charlton KE, Senekal M. Application and interpretation of multiple statistical tests to evaluate validity of dietary intake assessment methods. Nutr J. 2015;14(1). Available from: http://dx.doi.org/10.1186/s12937-015-0027-y

34. Mumu SJ, Merom D, Ali L, Fahey PP, Hossain I, Rahman AKMF, et al. Validation of a food frequency questionnaire as a tool for assessing dietary intake in cardiovascular disease research and surveillance in Bangladesh. Nutr J. 2020;19(1). Available from: http://dx.doi.org/10.1186/s12937-020-00563-7

35. Oviedo-Solís CI, Monterrubio-Flores EA, Cediel G, Denova-Gutiérrez E, Barquera S. Relative validity of a semi-quantitative food frequency questionnaire to estimate

dietary intake according to the NOVA classification in Mexican children and adolescents. J Acad Nutr Diet. 2022;122(6):1129–40. Available from: http://dx.doi.org/10.1016/j.jand.2021.11.002

36. Bland JM, Altman DG. Measuring agreement in method comparison studies. Stat Methods Med Res. 1999;8(2):135–60. Available from: http://dx.doi.org/10.1191/096228099673819272

37. Bland JM, Altman DG. Statistical methods for assessing agreement between two methods of clinical measurement. Int J Nurs Stud. 2010;47(8):931–6. Available from: http://dx.doi.org/10.1016/j.ijnurstu.2009.10.001

38. Sierra-Ruelas É, Bernal-Orozco MF, Macedo-Ojeda G, Márquez-Sandoval YF, Altamirano-Martínez MB, Vizmanos B. Validation of semiquantitative FFQ administered to adults: a systematic review. Public Health Nutr. 2021;24(11):3399–418. Available from: http://dx.doi.org/10.1017/s1368980020001834

39. Collins RA, Baker B, Coyle DH, Rollo ME, Burrows TL. Dietary assessment methods in military and veteran populations: A scoping review. Nutrients. 2020;12(3):769. Available from: http://dx.doi.org/10.3390/nu12030769

40. Mertens E, Kuijsten A, Geleijnse JM, Boshuizen HC, Feskens EJM, van’t Veer P. FFQ versus repeated 24-h recalls for estimating diet-related environmental impact. Nutr J. 2019;18(1). Available from: http://dx.doi.org/10.1186/s12937-018-0425-z

41. Streppel MT, de Vries JHM, Meijboom S, Beekman M, de Craen AJM, Slagboom PE, et al. Relative validity of the food frequency questionnaire used to assess dietary intake in the Leiden Longevity Study. Nutr J. 2013;12(1). Available from: http://dx.doi.org/10.1186/1475-2891-12-75

42. Evans S, Ashmore C, Daly A, Jackson R, Pinto A, MacDonald A. Validation of a low-protein semi-quantitative food frequency questionnaire. Nutrients. 2022;14(8):1595. Available from: http://dx.doi.org/10.3390/nu14081595

43. Pedisić Z, Bender DV, Duraković MM. Construction and reproducibility of a questionnaire aimed for evaluation of dietary habits in physically active individuals. Coll Antropol. 2008;32(4):1069–77.

44. Tollosa D, Van Camp J, Huybrechts I, Huybregts L, Van Loco J, De Smet S, et al.

Validity and reproducibility of a food frequency questionnaire for dietary factors related to colorectal cancer. Nutrients. 2017;9(11):1257. Available from: http://dx.doi.org/10.3390/nu9111257

45. Barrat E, Aubineau N, Maillot M, Derbord É, Barthes P, Lescuyer JF, et al. Repeatability and relative validity of a quantitative food-frequency questionnaire among French adults. Food Nutr Res. 2012;56(1):18472. Available from: http://dx.doi.org/10.3402/fnr.v56i0.18472

46. Zhang H, Qiu X, Zhong C, Zhang K, Xiao M, Yi N, et al. Reproducibility and relative validity of a semi-quantitative food frequency questionnaire for Chinese pregnant women. Nutr J. 2015;14(1). Available from: http://dx.doi.org/10.1186/s12937-015-0044-x

47. Tang Y, Liu Y, Xu L, Jia Y, Shan D, Li W, et al. Validity and reproducibility of a revised semi-quantitative food frequency questionnaire (SQFFQ) for women of age-group 12-44 years in Chengdu. J Health Popul Nutr. 2015;33(1):50–9.

48. El Kinany K, Garcia-Larsen V, Khalis M, Deoula MMS, Benslimane A, Ibrahim A, et al. Adaptation and validation of a food frequency questionnaire (FFQ) to assess dietary intake in Moroccan adults. Nutr J. 2018;17(1). Available from: http://dx.doi.org/10.1186/s12937-018-0368-4

49. Ding Y, Li F, Hu P, Ye M, Xu F, Jiang W, et al. Reproducibility and relative validity of a semi-quantitative food frequency questionnaire for the Chinese lactating mothers. Nutr J. 2021;20(1). Available from: http://dx.doi.org/10.1186/s12937-021-00678-5

50. Burkholder-Cooley NM, Rajaram SS, Haddad EH, Oda K, Fraser GE, Jaceldo-Siegl K. Validating polyphenol intake estimates from a food-frequency questionnaire by using repeated 24-h dietary recalls and a unique method-of-triads approach with 2 biomarkers. Am J Clin Nutr. 2017;105(3):685–94. Available from: http://dx.doi.org/10.3945/ajcn.116.137174

51. Whitton C, Ho J, Tay Z, Rebello S, Lu Y, Ong C, et al. Relative validity and reproducibility of a food frequency questionnaire for assessing dietary intakes in a multi-ethnic Asian population using 24-h dietary recalls and biomarkers. Nutrients. 2017;9(10):1059. Available from: http://dx.doi.org/10.3390/nu9101059

52. Alawadhi B, Fallaize R, Franco RZ, Hwang F, Lovegrove J. Web-based dietary intake estimation to assess the reproducibility and relative validity of the EatWellQ8 food frequency questionnaire: Validation study. JMIR Form Res. 2021;5(3): e13591. Available from: http://dx.doi.org/10.2196/13591

53. Yanagisawa A, Sudo N, Amitani Y, Caballero Y, Sekiyama M, Mukamugema C, et al. Development and validation of a data-based food frequency questionnaire for adults in eastern rural area of Rwanda. Nutr Metab Insights. 2016;9: NMI.S38374. Available from: http://dx.doi.org/10.4137/nmi.s38374

54. Morel S, Portolese O, Chertouk Y, Leahy J, Bertout L, Laverdière C, et al. Development and relative validation of a food frequency questionnaire for French-Canadian adolescent and young adult survivors of acute lymphoblastic leukemia. Nutr J. 2018;17(1). Available from: http://dx.doi.org/10.1186/s12937-018-0355-9

55. Fallaize R, Forster H, Macready AL, Walsh MC, Mathers JC, Brennan L, et al. Online dietary intake estimation: Reproducibility and validity of the Food4Me food frequency questionnaire against a 4-day weighed food record. J Med Internet Res. 2014;16(8): e190. Available from: http://dx.doi.org/10.2196/jmir.3355

56. Cheng Z, Shuai P, Qiao Q, Li T. Validity and reliability of a simplified food frequency questionnaire: a cross sectional study among physical health examination adults in southwest region of China. Nutr J. 2020;19(1). Available from: http://dx.doi.org/10.1186/s12937-020-00630-z

57. Sasaki K, Sunami A, Yoshimura Y, Katsumata Y, Kisara K, Yokoyama Y, et al. Validity of a semi-quantitative food frequency questionnaire based on food groups and cooking with a protein supplement in Japanese collegiate athletes. J Phys Fit Sports Med. 2018;7(2):131–42. Available from: http://dx.doi.org/10.7600/jpfsm.7.131

58. Zaragoza-Martí A, Cabañero-Martínez MJ, Hurtado-Sánchez JA, Laguna-Pérez A, Ferrer-Cascales R. Evaluation of Mediterranean diet adherence scores: a systematic review. BMJ Open. 2018;8(2): e019033. Available from: http://dx.doi.org/10.1136/bmjopen-2017-019033

59. Archer E, Marlow ML, Lavie CJ. Controversy and debate: Memory-Based Methods Paper 1: the fatal flaws of food frequency questionnaires and other memory-based dietary assessment methods. J Clin Epidemiol. 2018; 104:113–24. Available from:

http://dx.doi.org/10.1016/j.jclinepi.2018.08.003

60. Loy SL, Marhazlina M, Nor AY, Hamid JJM. Development, validity and reproducibility of a food frequency questionnaire in pregnancy for the Universiti Sains Malaysia birth cohort study. Malays J Nutr. 2011;17(1):1–18.

61. Goode JP, Smith KJ, Kilpatrick M, Breslin M, Oddy WH, Dwyer T, et al. Retrospectively estimating energy intake and misreporting from a qualitative food frequency questionnaire: An example using Australian cohort and national survey data. Front Nutr. 2021;8. Available from: http://dx.doi.org/10.3389/fnut.2021.624305

62. Shahar S, Shahril MR, Abdullah N, Borhanuddin B, Kamaruddin MA, Yusuf NAM, et al. Development and relative validity of a semiquantitative food frequency questionnaire to estimate dietary intake among a multi-ethnic population in the Malaysian Cohort project. Nutrients. 2021;13(4):1163. Available from: http://dx.doi.org/10.3390/nu13041163

63. Ishikawa-Takata K, Okamoto K, Taguchi M. Development and validation of a food frequency questionnaire for Japanese athletes (FFQJA). J Int Soc Sports Nutr. 2021;18(1). Available from: http://dx.doi.org/10.1186/s12970-021-00433-5

64. Cui Q, Xia Y, Wu Q, Chang Q, Niu K, Zhao Y. A meta-analysis of the reproducibility of food frequency questionnaires in nutritional epidemiological studies. Int J Behav Nutr Phys Act. 2021;18(1). Available from: http://dx.doi.org/10.1186/s12966-020-01078-4

65. Sunami A, Sasaki K, Suzuki Y, Oguma N, Ishihara J, Nakai A, et al. Validity of a semi-quantitative food frequency questionnaire for collegiate athletes. J Epidemiol. 2016;26(6):284–91. Available from: http://dx.doi.org/10.2188/jea.je20150104

66. Robertson C, Conway R, Dennis B, Yarnell J, Stamler J, Elliott P. Attainment of precision in implementation of 24h dietary recalls: INTERMAP UK. Br J Nutr. 2005;94(4):588–94. Available from: http://dx.doi.org/10.1079/bjn20051543

403

Figure 1

Click here to access/download;Figure;Fig 1.tif.tif

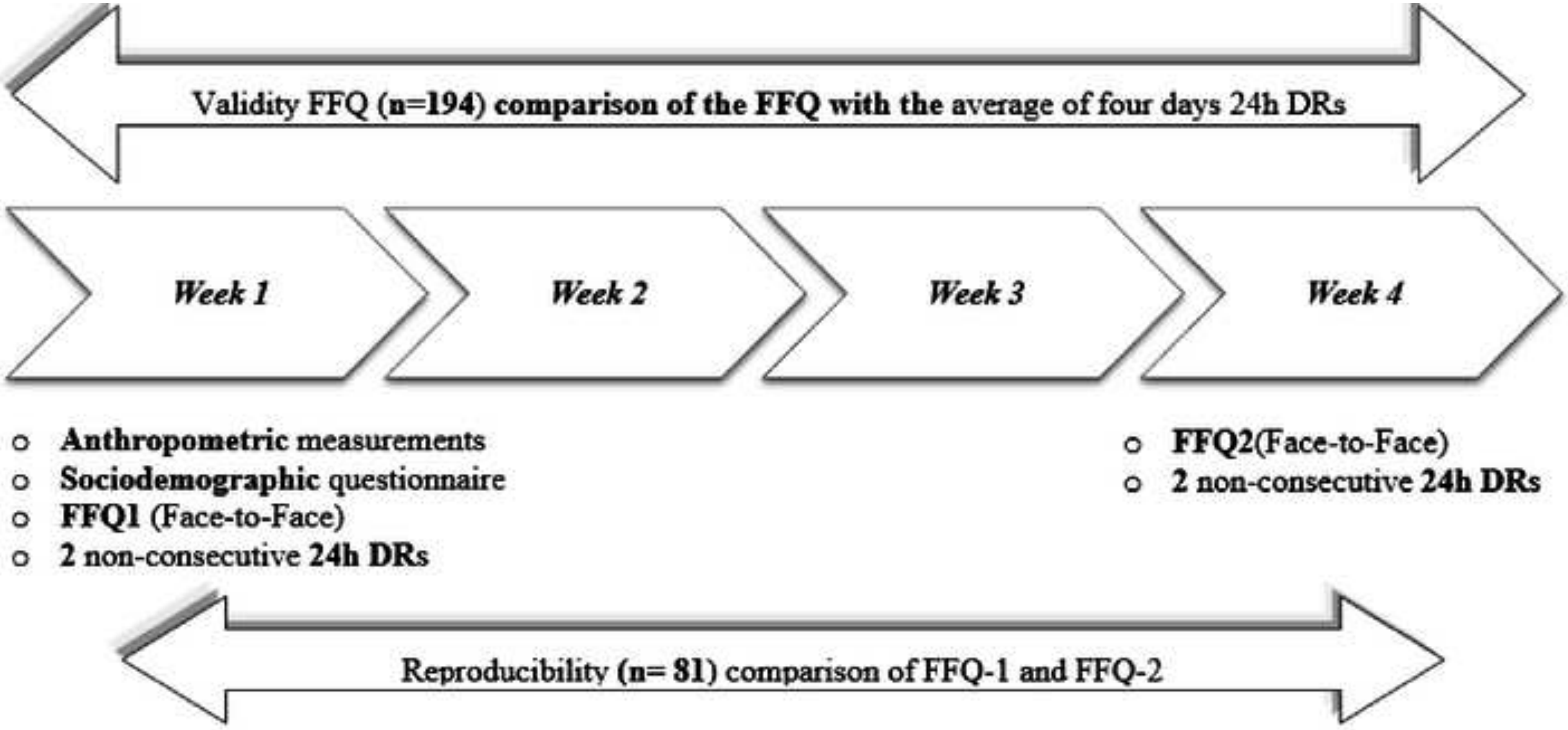

Fig 1.Flowchart of the validation and reproducibility study among Lebanese athletes

Figure 2

Click here to access/download;Figure;Fig 2.tif.tif

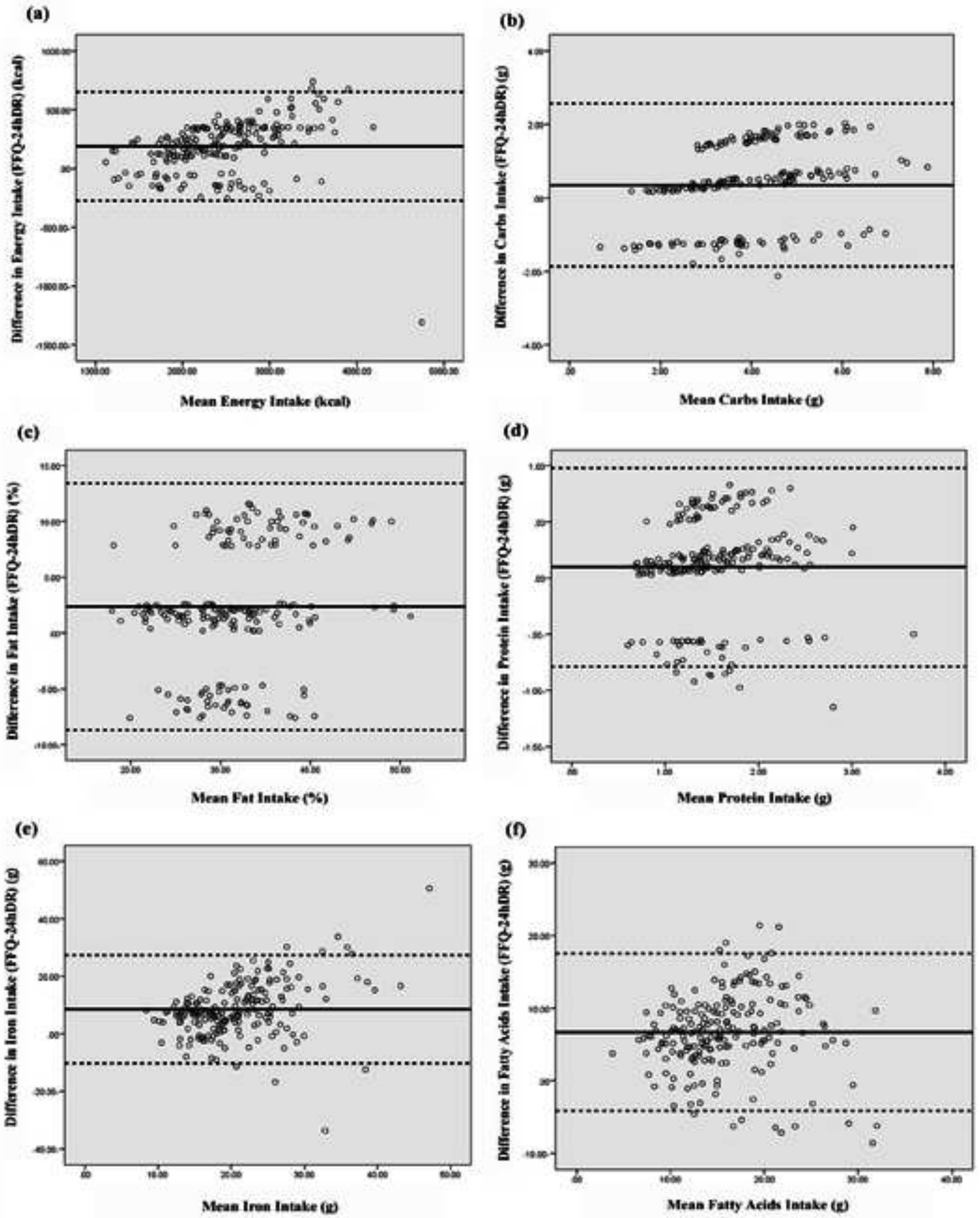

Fig2. Bland-Altman plots represent the agreement between the average of nutrient intakes measured from FFQ1 and the average of four 24hDRs. (a) Bland-Altman analysis for energy intake; (b) Bland-Altman analysis for carbs intake; (c) Bland-Altman analysis for fat intake; (d) Bland-Altman analysis for protein intake; (e) Bland-Altman analysis for iron intake; (f) Bland-Altman analysis for fatty acids intake.

Supporting Information 1

Click here to access/download
Supporting Information
S1.xlsx

Supporting Information 2

Click here to access/download
Supporting Information
S2.xlsx

Click here to access/download
Supporting Information
S3.docx
