# Supplementary material for: Validity and reproducibility of a food frequency questionnaire to determine dietary intakes among Lebanese athletes
Source: PLoS One. 2024 Oct 23;19(10):e0311617. doi: 10.1371/journal.pone.0311617 (PMC11498687; doi:10.1371/journal.pone.0311617)
Supplement: Attachment — Submitted filename: Comment.docx [file pone.0311617.s005.docx]

1. Authors should refer to the methods used to measure validity and reliability in the abstract method.
2. The authors stated in the abstract results that the amount of ICC is excellent. According to various sources, ICC value above 0.9 is excellent, 0.75 to 0.9 is good, 0.5 to 0.75 is average, and below 0.5 is poor. Therefore, the authors should not introduce the value of 0.688 or 0.739 or 0.870 as excellent.
3. The logic of calculating the sample size of 194 athletes should be explained in the method section.
4. In lines 196-202, the authors should not use the term excellent reproducibility because not all ICCs are above 0.9.
5. As an educational note, in scientific writing all tables and figures should be moved to the end of the last reference.
6. In lines 206 to 214, the author reports Spearman's correlation coefficient. The value of this index is between -1 and +1. Please state the reporting range of Spearman's correlation coefficient on this scale.
7. I do not understand the concept of seasonality bias in line 258 of the discussion. Please explain it.
8. In the discussion of the article, the author should not simply compare his findings with others. In the discussion, they should mention the reasons for the differences between their findings and others. Please consider this point in rewriting the discussion.
